# Phytochemical Composition, Anti-Inflammatory and ER Stress-Reducing Potential of *Sambucus ebulus* L. Fruit Extract

**DOI:** 10.3390/plants10112446

**Published:** 2021-11-12

**Authors:** Oskan Tasinov, Ivayla Dincheva, Ilian Badjakov, Yoana Kiselova-Kaneva, Bistra Galunska, Ruben Nogueiras, Diana Ivanova

**Affiliations:** 1 Department of Biochemistry, Molecular Medicine and Nutrigenomics, Medical University of Varna, 84B Tzar Osvoboditel Blvd., 9002 Varna, Bulgaria; yoana.kiselova@mu-varna.bg (Y.K.-K.); bistra.galunska@gmail.com (B.G.); divanova@mu-varna.bg (D.I.); 2 AgroBioInstitute, Agricultural Academy, 8 Dr. Tsankov Blvd., 1164 Sofia, Bulgaria; ivadincheva@yahoo.com (I.D.); ibadjakov@gmail.com (I.B.); 3 Center for Research in Molecular Medicine and Chronic Diseases (CiMUS), Department of Physiology, University of Santiago de Compostela-Instituto de Investigación Sanitaria, 15782 Santiago de Compostela, Spain; ruben.nogueiras@usc.es; 4 CIBER Fisiopatología de la Obesidad y Nutrición (CIBERobn), 15706 Santiago de Compostela, Spain

**Keywords:** *Sambucus ebulus* L., phytochemical composition, anti-inflammatory, ER stress, lipopolysaccharides, macrophages

## Abstract

*Sambucus ebulus* L. (SE) fruits are used for their immunostimulation, hematopoietic and antiviral potential. Recently, we focused on analyzing the mechanism underlying SE fruit aqueous extract’s (FAE) immunomodulation and anti-inflammatory activities, with attention to its endoplasmic reticulum (ER) stress-reducing potential. J774A.1 macrophages were treated with SE FAE alone or in conditions of lipopolysaccharides (LPS) stimulation. Using GC–MS and LC–MS/MS, its phytochemical composition was analyzed. To measure transcription and protein levels, we used qPCR and Western blot, respectively. The prevailing phytochemicals in SE FAE were hydroxycinnamic acids, proanthocyanidins and anthocyanins. The content of some amino acids, organic acids, alcohols, fatty acids and esters were newly reported. Extracts exerted an immunostimulation potential by stimulating *IL-6, TNFα, Ccl2, COX2* and *iNOS* transcription, without inducing ER stress. SE FAE suppressed the LPS-induced transcription of inflammation related genes (*IL-1β, IL-6, TNFα, Ccl2, Icam-1, Fabp4, COX2, iNOS, Noxo1, IL-1ra, Sirt-1*) and reduced the protein levels of iNOS, peIF2α, ATF6α and CHOP. The effects were comparable to that of salicylic acid. SE suppresses LPS-stimulated inflammatory markers on the transcription and translation levels. Targeting ER stress is possibly another mechanism underlying its anti-inflammatory potential. These findings reveal the potential of SE fruits as a beneficial therapeutic of inflammation and ER stress-related pathological conditions.

## 1. Introduction

Traditional medicine is a good source of knowledge about therapeutics, which are consequently researched and successfully implicated in modern pharmaceutical preparations. *Sambucus ebulus* L. (SE), also known as dwarf elder or dwarf elderberry, is a widely used as wound-healing, anti-nociceptive, anti-rheumatoid, anti-influenza, antibacterial and diuretic medicinal plant in Bulgaria, Turkey, Iran, Lebanon, Romania and Bosnia–Herzegovina [1,2,3,4,5]. Fresh fruits, jam, tea or decoction of SE fruits are used as immunostimulating and hematopoietic herbal preparations, as well as for the treatment of rheumatoid arthritis and gastrointestinal disorders [1,2,6]. The number of modern studies focusing on SE biological activities are growing, but there is still insufficient knowledge regarding molecular mechanisms of action of fresh or dry fruits and various fruit extracts.

Only ripe fruits are used in traditional medicine recipes and the chemical content varies depending on the types of the extract [3,7]. Data from phytochemical analyses in the literature reveal that SE fruits are high in polyphenolics, especially anthocyanins and proantchocyanidins, phenolic acids, hydroxycinnamic acids, flavonol glycosides, as well as organic acids, tannins, pectins, resins, vitamin C, volatile substances (eugenol, valeric acid, citronellal etc.), amino acids (including some essential ones), and plant sterols [3,7,8,9,10,11,12,13,14,15,16]. Many chromatographic analyses of SE fruit extracts have been carried out up to date, and, still, the information about the presence of certain specific organic compounds remains unclear, especially with regard to soil characteristics, variety of extragents used for sample preparation. Therefore, a detailed phytochemical analysis could be useful, especially in examining the molecular mechanisms of SE fruits on human health.

Numerous studies have established the strong in-vitro antioxidant activity of SE fruit extracts, analyzing its iron chelating, NO radical scavenging, and ABTS cation radical decolorization activity, and their interrelations with polyphenolic and anthocyanin content [3,7,8]. The presence of different functional groups in polyphenolics and organic acids found in the tested SE fruit extracts is considered to determine, to a great extent, their antioxidant and anti-inflammatory activities. In oxidatively challenged 3T3-L1 preadipocytes, SE fruit aqueous extract (FAE) acts as modulator of antioxidant genes’ transcription [17]. In macrophages treated with ethanol- or lipopolysaccharides (LPS), SE FAE suppresses the ethanol- and LPS-stimulated transcription of glutamate–cysteine ligase, glutathione peroxidase and nuclear factor kappa B (NFκB) [9,18]. Acetone extracts, hydrophilic and anthocyanin-rich fractions of SE fruits possessing high in-vitro antioxidant activity protect macrophages from the oxidative stress-mediated cytotoxicity caused by *tert*-Butyl hydroperoxide [19]. Ethyl acetate fraction of SE fruits possesses cytoprotective and anti-inflammatory activity reducing ethanol-induced cell death, proinflammatory gene transcription in macrophages [9]. Methanolic extracts of SE fruits reduce carrageenan-induced paw edema in rats [20]. Others describe the antiemetic, neuroprotective and anti-herpes-simplex-virus activities of SE fruit extracts [12,21].

In an intervention study on healthy adult volunteers, SE fruit tea enhances serum antioxidant potential, improves lipid profile [22], decreases serum CRP, IL-1β, leptin and adiponectin levels [23], thus indicating an immune- and fat metabolism-modulating activity. A clinical trial reported the effectiveness of SE fruit ethanol extract for the treatment of paederus dermatitis, proving its anti-inflammatory and wound healing potential [24].

LPS-stimulated macrophages are widely used in-vitro models for testing anti-inflammatory activity of medicinal plant extracts. The macrophages are source of a variety of pro-inflammatory cytokines, chemokines, and may act in a paracrine and endocrine mode. In low grade inflammation, such as in adiposity, where the activation of chemokine release is associated with macrophage recruitment and unlocking a self-feeding inflammatory process that leads to such complications as insulin resistance and related atherosclerosis [25]. The released cytokines and chemokines, such as TNFα, IL-6, IL-1β, NO, as a product of iNOS, activate signaling pathways mediated by Jun N-terminal kinase (JNK), the inhibitor of κB-kinase (IKK)β and other serine kinases [25,26,27,28], and resulting in NFκB activation. The latter stimulates the transcription of pro-inflammatory genes [29].

Along with the protein synthesis, endoplasmic reticulum (ER) plays an important role in sensing nutrients and responds to different stress conditions by activating the unfolded protein response and subsequently implicating it into insulin resistance and cardiovascular diseases [30,31]. ER stress can promote inflammation, and vice versa [32,33]. ER stress-related inflammation could be mediated by iNOS [34]. Therefore, the enzyme iNOS as a cross point of inflammation and ER stress could be a possible therapeutic target.

There are data that ER stress and inflammation in different pathological conditions could be reduced by compounds such as resveratrol [35,36], epigallocatechin gallate [37] and proanthocyanidins found in herbal extracts [38]. SE fruits, being rich polyphenolics, anthocyanins and stilbenes, could be effective in combating ER stress and inflammation.

We aimed to analyze the phytochemical composition of SE FAE and to test its immune- and ER stress-modulating potential in a model of unstimulated and LPS-challenged J774A.1 mouse macrophages. The phytochemical analysis of SE FAE revealed the presence of numerous compounds with anti-inflammatory and ER stress-reducing activity. For first time it was established that the transcription-modulating effect of SE FAE on inflammatory cytokines, chemokines, and enzymes in non-stimulated macrophages. In LPS-challenged macrophages, SE FAE suppresses the translation of iNOS and ER stress-related proteins.

## 2. Results

### 2.1. Phytochemical Content and Composition

Among the phytochemical compounds identified in the tested SE FAE 15 amino acids (AAs), 10 organic acids (OAs), 36 sugar acids and alcohols, 25 mono-, di- and trisaccharides, 13 fatty acids (saturated and unsaturated) and their esters (Table 1), and 38 phenolic compounds were detected and quantified (Table 2).

#### 2.1.1. Polar Compounds

The most abundant AAs were L-glutamine (18.20% of AAs content) followed by L-proline (15.84% of AAs), L-aspartic acid (12.92% of AAs), DL-ornithine (9.78% of AAs). Six of all fifteen identified AAs were essential (Val, Leu, Ile, Thr, Phe and Lys). Among them phenylalanine was found to be in highest concentration (10.25 ± 0.75 µg/mL), followed by isoleucine (8.48 ± 0.49 µg/mL) and leucine (8.06 ± 0.56 µg/mL) (Table 1). All essential AA comprise 30% of all detected AA content in SE extract.

Among the identified polar OAs, pyroglutamic acid (30.39% of OAs content) and isocitric acid (16.37% of OAs) are found to be in highest concentrations (Table 1). Sorbitol and its 6-phosphate are pre-dominant (92.58 ± 3.24 µg/mL, 34.47% of alcohols) alcohols in the SE FAE, followed by glycerol and its 3-phosphate (60.84 ± 3.35 µg/mL, 22.65% of alcohols) and arabinitol (34.65 ± 2.43 µg/mL, 12.90% of alcohols). Glucuronic and galacturonic acid isomers and glyceric acid were among the prevailing sugar acids (Table 1).

Dominating saccharides were galactose and its 6-phosphate form (57.30 µg/mL), followed by glucose and its 6-phosphate form (52.13 µg/mL) and sorbose (49.46 µg/mL). Sucrose (45.36 µg/mL) was the prevailing disaccharide in the extract. In total, the amount of saccharides was 411.11 µg/mL (Table 1).

Among the tested fatty acids and fatty esters, we found the highest concentration for octadecadienoic acid (15.65 ± 1.41 µg/mL, 18.41% of fatty acids) and for beta-sitosterol (15.22 ± 1.37 µg/mL, 40.43% of esters and sterols) (Table 1).

#### 2.1.2. Polyphenolic Content

*S. ebulus* L. fruits are a rich source of polyphenolics especially anthocyanins, proanthocyanidins and phenolic acids (Table 2). Cyanidin-3-O-galactoside (382.15 µg/mL, 74.43% of anthocyanins) was at highest concentration among anthocyanins and epicatechin was the major proanthocyanidin (322.37 µg/mL). Among proanthocyanidin polymers dominate epicatechin dimers (688.42 µg/mL) and trimers (915.79 µg/mL) comprising 11.79% and 15.68%, respectively, of all detected polyphenolics in our samples. All together anthocyanins and proanthocyanidins were 1966.76 µg/mL representing 33.67% of all analyzed polyphenols. Trans-Resveratrol-3-O-glucoside (51.93 µg/mL) was the only detected stilbene. Dominating hydroxycinnamic acids found in *S. ebulus* fruits are neochlorogenic and chlorogenic acid, followed by 3-O-p-coumaroylquinic acid and feruloylquinic acid. In total, the amount of hydroxycinnamic acids in the tested SE extract was 3005.02 µg/mL (300.5 mg/g DW) and represented 51.45% of all detected polyphenols. Hyperoside was one the major flavonol detected in our samples.

### 2.2. Investigation of Inflammation Related Biomarkers in a Model of LPS-Stimulated J774A.1 Macrophages

Aiming to study the anti-inflammatory action of the aqueous extract of dwarf elderberry under conditions of LPS-stimulated inflammatory response in J774A.1 mouse macrophages, the transcriptional levels of genes coding for proteins mediating and involved in the inflammatory process as well as the translation levels of iNOS were analyzed. Macrophage cells were pre-treated with increasing concentrations of 2.5%, 5% and 10% *v*/*v* (0.25 mg DW/mL, 0.5 mg DW/mL, 1 mg DW/mL respectively) SE FAE or salicylic acid (SA) for 24 h followed by LPS stimulation for an additional 24 h, and included respective control treatments. It was previously reported that the concentrations of extract used in recent experiment are non-toxic for the J774A.1 cell line [18]. The analyzed genes included interleukin 1 beta (*IL-1β*), interleukin 6 (*IL-6*), tumor necrosis factor alpha (*TNFα*), monocyte chemoattractant protein-1 (*MCP-1*, chemokine nomenclature: C–C motif chemokine ligand 2 (*Ccl2*)), intercellular adhesion molecule-1 (*Icam1*), fatty acid binding protein 4 (*Fabp4*, adipocyte protein 2 (*aP2*), prostaglandin-endoperoxide synthase 2 (*Ptgs2*, cyclooxygenase-2 (*COX2*)), inducible NO synthase (*iNOS*), NADPH oxidase organizer 1 (*Noxo1*), interleukin 1 beta receptor antagonist (*IL-1ra*) and sirtuin 1 (*Sirt-1*). The intracellular iNOS protein levels were analyzed as well.

#### 2.2.1. The Effect of LPS-Stimulation on Inflammation Related Biomarkers in J774A.1 Macrophages

**Figure 1:**
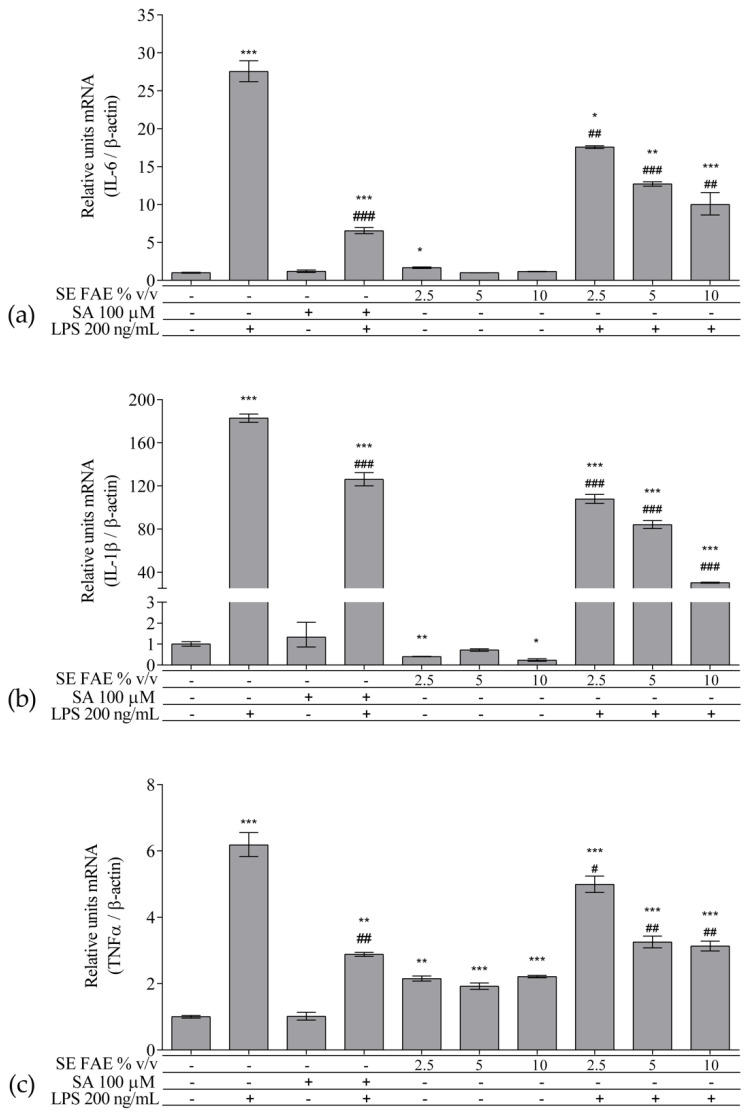
Changes in mRNA levels of *IL-1β* (**a**), *IL-6* (**b**), and *TNFα* (**c**) in J774A.1 mouse macrophages pre-treated with increasing concentrations (2.5%, 5%, 10% *v*/*v*) of SE FAE or with SA for 24 h and subsequently stimulated or not with LPS. Results were obtained using qPCR technique. Data are presented as mean ± SEM. Legend: SE FAE–*Sambucus ebulus* L. fruit aqueous extract; SA–100 μM salicylic acid; LPS–200 ng/mL lipopolysaccharides. * *p* < 0.05, ** *p* < 0.01, *** *p* < 0.001 vs. untreated cells; # *p* < 0.05, ## *p* < 0.01, ### *p* < 0.001 vs. LPS.

**Figure 2:**
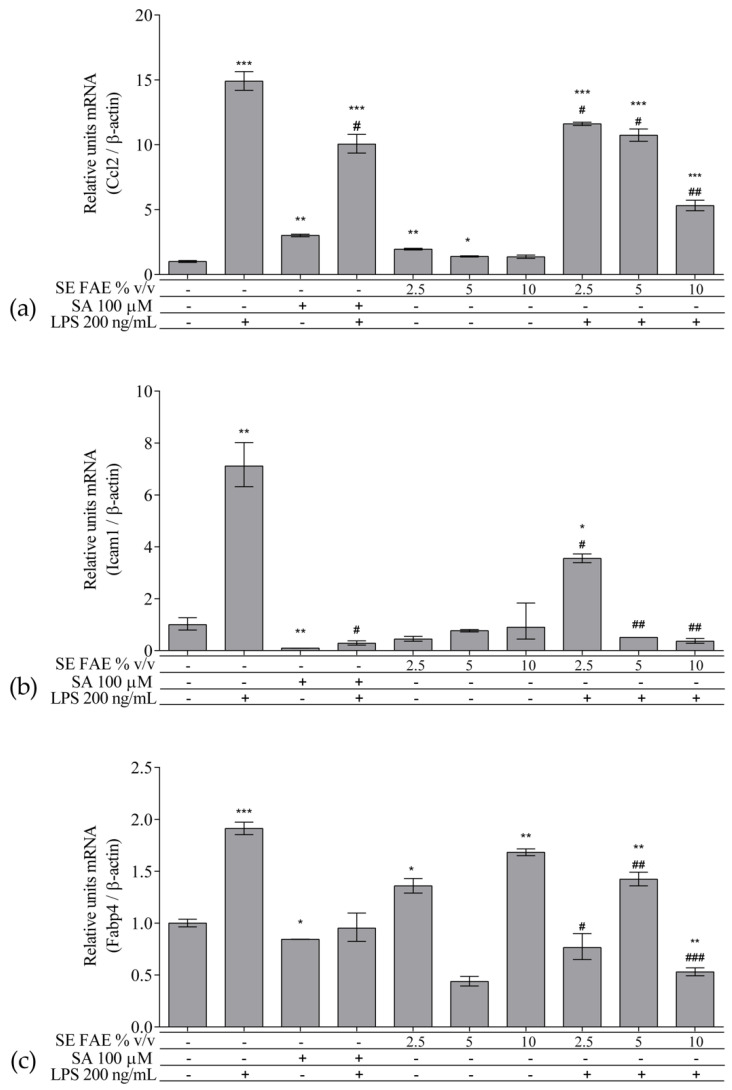
Changes in mRNA levels of *Ccl2* (**a**), *Icam1* (**b**), and *Fabp4* (**c**) in J774A.1 mouse macrophages pre-treated with increasing concentrations (2.5%, 5%, 10% *v*/*v*) of SE FAE or with SA for 24 h and subsequently stimulated or not with LPS. Results were obtained using qPCR technique. Data are presented as mean ± SEM. Legend: SE FAE–*Sambucus ebulus* L. fruit aqueous extract; SA–100 μM salicylic acid; LPS–200 ng/mL lipopolysaccharides. * *p* < 0.05, ** *p* < 0.01, *** *p* < 0.001 vs. untreated cells; # *p* < 0.05, ## *p* < 0.01, ### *p* < 0.001 vs. LPS.

**Figure 3:**
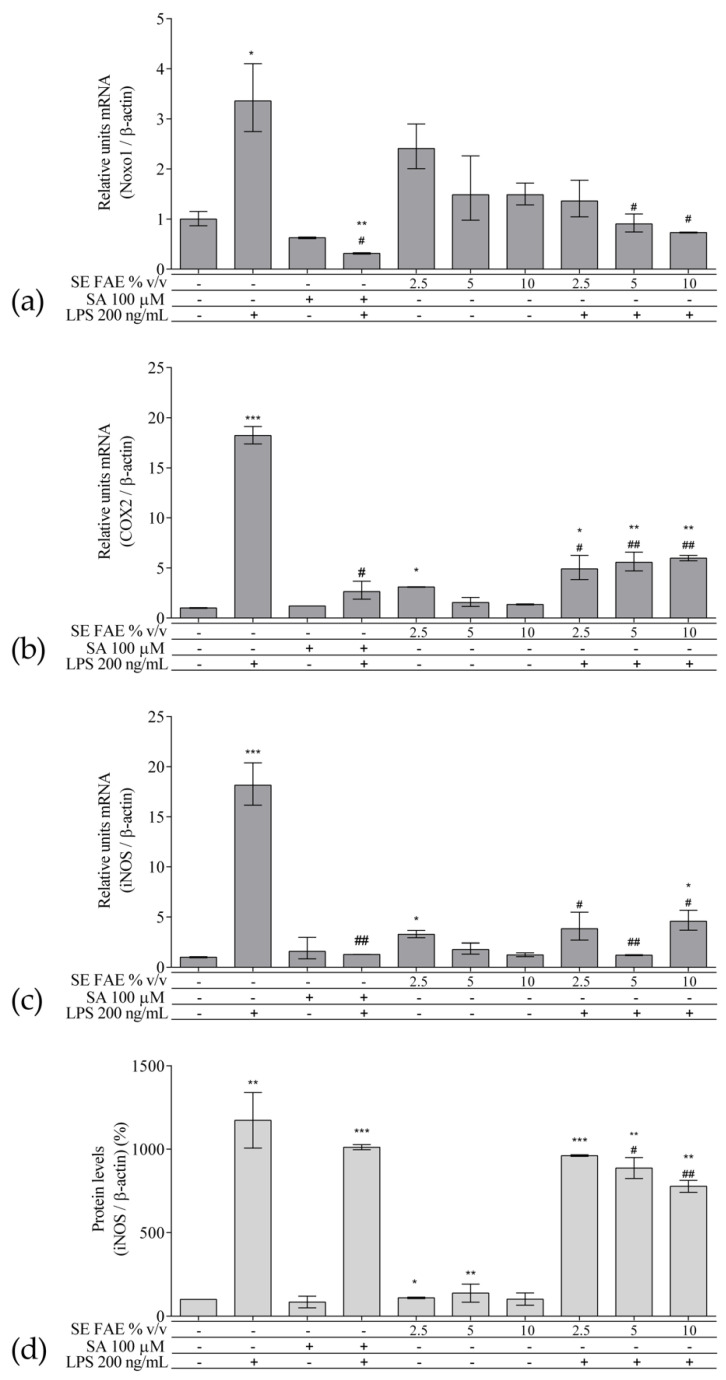
Changes in mRNA levels of *COX2* (**a**), *iNOS* (**b**), *Noxo1* (**c**), and of protein levels of iNOS (**d**) in J774A.1 mouse macrophages pre-treated with increasing concentrations (2.5%, 5%, 10% *v*/*v*) of SE FAE or with SA for 24 h and subsequently stimulated or not with LPS. Results were obtained using qPCR ((**a**), (**b**) and (**c**)) or western blot technique (**d**). Data are presented as mean ± SEM. Legend: SE FAE–*Sambucus ebulus* L. fruit aqueous extract; SA–100 μM salicylic acid; LPS–200 ng/mL lipopolysaccharides. * *p* < 0.05, ** *p* < 0.01, *** *p* < 0.001 vs. untreated cells; # *p* < 0.05, ## *p* < 0.01 vs. LPS treatment.

**Figure 4:**
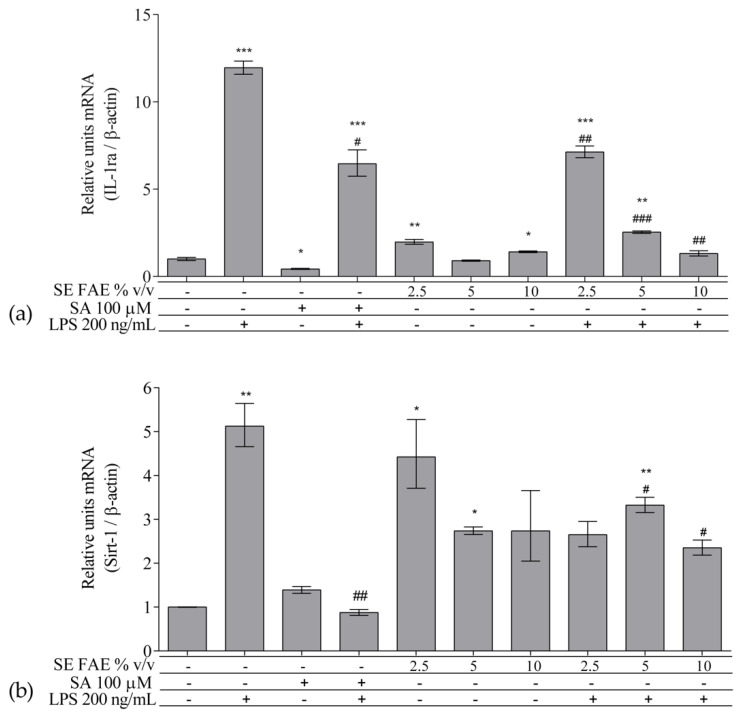
Changes in mRNA levels of *IL-1ra* (**a**) and of *Sirt-1* (**b**) in J774A.1 mouse macrophages pre-treated with increasing concentrations (2.5%, 5%, 10% *v*/*v*) of SE FAE or with SA for 24 h and subsequently stimulated or not with LPS. Results were obtained using qPCR technique. Data are presented as mean ± SEM. Legend: SE FAE–*Sambucus ebulus* L. fruit aqueous extract; SA–100 μM salicylic acid; LPS–200 ng/mL lipopolysaccharides. * *p* < 0.05, ** *p* < 0.01, *** *p* < 0.001 vs. untreated cells; # *p* < 0.05, ## *p* < 0.01, ### *p* < 0.001 vs. LPS treatment.

As an inflammatory agent, LPS increased transcription levels of *IL-1β, IL-6, TNFα, Ccl2, Icam1* and *Fabp4* by fold-changes of 182 (*p* < 0.001), 27 (*p* < 0.001), 6 (*p* < 0.001), 14.9 (*p* < 0.001), 7 (*p* < 0.01) and 1.9 (*p* < 0.001), respectively (Figure 1a–c and Figure 2a–c). Similarly, LPS-stimulated transcription of *COX2, iNOS*, and of *Noxo1* by 18 (*p* < 0.001), 18 (*p* < 0.001), 3.4 (*p* < 0.05) folds, respectively, and of iNOS protein levels as well by 11.7 (*p* < 0.01) folds (Figure 3a–d). Concerning anti-inflammatory genes’ expression, we observed a 12- (*p* < 0.001) and 5-fold (*p* < 0.01) increase for *IL-1ra* and *Sirt-1*, respectively (Figure 4a,b).

#### 2.2.2. The Effect of SE FAE on Inflammation-Related Biomarkers in Non-Stimulated J774A.1 Macrophages

When applied alone, 2.5% *v*/*v* and 10% *v*/*v* SE FAE slightly reduced the gene expression of *IL-1β* by 60% (*p* < 0.01) and 77% (*p* < 0.05), respectively, as compared to untreated cells (Figure 1a). While 2.5% *v*/*v* of herbal extract induced the gene expression of *IL-6* (by 67%, *p* < 0.05), *TNFα* (by 115%, *p* < 0.01), *Ccl2* (by 95%, *p* < 0.01), and *Fabp4* (by 36%, *p* < 0.05) (Figure 1b,c and Figure 2a,c). The higher concentration of SE FAE (5% extract) in culture media stimulated transcription of *TNFα* (by 92%, *p* < 0.001) and of *Ccl2* (by 39%, *p* < 0.05) (Figure 1c and Figure 2a), while the highest concentration (10% extract) induced transcription of *TNFα* (by 121%, *p* < 0.001) and of *Fabp4* (by 68%, *p* < 0.01) (Figure 1c and Figure 2c). SA, applied alone, similarly to SE FAE, it enhanced transcription levels of *Ccl2* (by 200%, *p* < 0.01), but in contrast with SE FAE, it slightly reduced those of *Icam1* (by 91%, *p* < 0.01) and of *Fabp4* (by 16%, *p* < 0.05) (Figure 2a–c), while no significant effects on *IL-1β, IL-6* and *TNFα* transcription levels were observed (Figure 1a–c).

The treatment with 2.5% *v*/*v* of SE FAE alone significantly induced the transcription levels of *COX2* (by 210%, *p* < 0.05) and of *iNOS* (by 230%, *p* < 0.05) and both 2.5% *v*/*v* and 5% *v*/*v* of the extract induced iNOS protein levels by 9% (*p* < 0.05) and by 38% (*p* < 0.01), respectively (Figure 3). No effect of SA alone was observed on the gene expression levels of all analyzed inflammation and phagocytosis-related enzymes (Figure 3).

SE FAE in concentrations of 2.5% *v*/*v* and 10% *v*/*v* induced the transcription levels of *IL-1ra* by 98% (*p* < 0.01) and 41% (*p* < 0.05), respectively (Figure 4a). In contrast, SA treatment reduced *IL-1ra* transcription by 57% (*p* < 0.05) (Figure 4a). Transcription of the so-called longevity gene *Sirt-1* was stimulated upon 2.5% *v*/*v* and 5% *v*/*v* SE FAE treatment by 343% (*p* < 0.05) and by 274% (*p* < 0.05), respectively (Figure 4b). There was no significant effect of SA applied alone on *Sirt-1* transcription levels (Figure 4b).

#### 2.2.3. The effect of SE FAE on Inflammation-Related Biomarkers in LPS-Stimulated J774A.1 Macrophages

In LPS-stimulated macrophages, the pre-treatment with all three increasing concentrations of SE FAE (2.5% *v*/*v*, 5% *v*/*v* and 10% *v*/*v*), as compared with LPS treatment, significantly reduced the transcription levels of *IL-1β, IL-6, TNFα, Ccl2*, and of *Icam1* with up to 83% (*p* < 0.001), 67.7% (*p* < 0.01), 49% (*p* < 0.01), 64% (*p* < 0.01), and 94.9% (*p* < 0.01), respectively (Figure 1a–c and Figure 2a,b). The effect followed a dose-dependent manner. Similarly, all concentrations reduced LPS-stimulated *Fabp4* mRNA levels, with stronger effect exerted by 2.5% *v*/*v* (by 60.2%, *p* < 0.05) and by 10% *v*/*v* (by 72.4%, *p* < 0.001) SE FAE (Figure 2c). Considering the effect of SE FAE alone on *Fabp4*, we may recognize that the same concentrations stimulating its gene expression (2.5% *v*/*v* and 10% *v*/*v*) are the ones exerting the stronger reducing effect in the case of LPS-stimulated cells.

Pre-treatment with the SA as a known anti-inflammatory compound, significantly reversed the LPS stimulation of all genes except *Fabp4*, as follows: *IL-1β* (31%, *p* < 0.001); *IL-6* (76%, *p* < 0.001); *TNFα* (53%, *p* < 0.01); *Ccl2* (32%, *p* < 0.05); *Icam1* (96%, *p* < 0.05) (Figure 1a–c and Figure 2a,b). The inhibitory effect of SE FAE on LPS-stimulated transcription of pro-inflammatory genes was similar to the effect of the positive control SA. In the case of *Icam1*, both the extract and the SA reduced the LPS-induced mRNA levels back to normal. When applied in highest concentration (10% *v*/*v*) the herbal extract had a reducing effect on the LPS-stimulated gene expression of *IL-1β*, *Ccl2*, and of *Fabp4*, which was even stronger than that of the SA.

SE FAE significantly inhibited the LPS-stimulated transcription levels of *COX2*, *iNOS* and of *Noxo1,* by up to 73% (*p* < 0.05), 93% (*p* < 0.01) and 78% (*p* < 0.05), respectively, and the protein levels of iNOS by up to 33% (*p* < 0.01) (Figure 3a–d). When SA was applied prior to LPS stimulation mRNA levels of *COX2*, *iNOS* and *Noxo1* were reduced by 85% (*p* < 0.05), 92.9% (*p* < 0.01), and by 90.7% (*p* < 0.05), respectively (Figure 3a–c). The effect shown by SE FAE was similar to that of SA and they both independently reduced LPS-stimulated transcription of *iNOS* and of *Noxo1* back to the normal levels. Pre-treatment with herbal extract showed a stronger iNOS mRNA- and protein levels-reducing effect than the SA did in LPS-challenged cells. 

Application of SE FAE suppressed the LPS-induced transcription of *IL-1ra* by up to 88.95% (*p* < 0.01) in a dose-dependent manner and that of *Sirt-1* by up to 54% (*p* < 0.05) (Figure 4). Similar effect was observed in the SA pre-treated cells, where LPS-induced *IL-1ra* and *Sirt-1* mRNA transcription levels were decreased by 46% (*p* < 0.05) and by 82% (*p* < 0.01), respectively (Figure 4). SE FAE exerted stronger reducing activity than that of SA on LPS-stimulated *IL-1ra* transcription, decreasing it to the normal levels. 

### 2.3. Investigation of ER Stress-Related Biomarkers in a Model of LPS-Stimulated J744A.1 Macrophages

**Figure 5:**
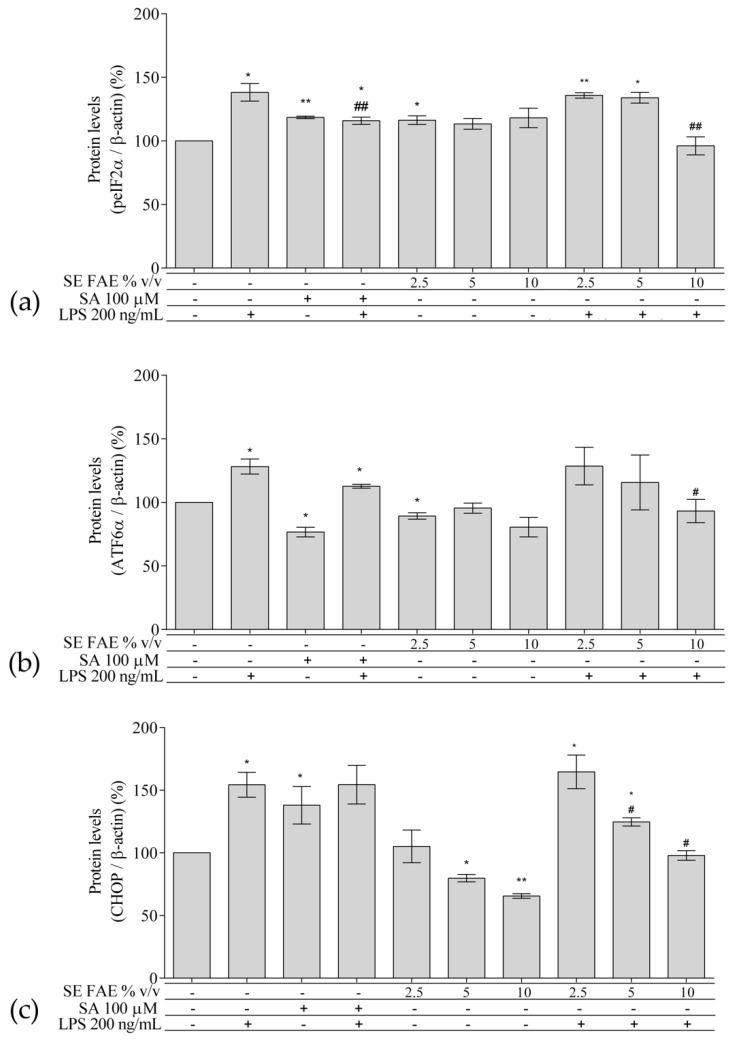
Changes in the protein levels of peIF2α (**a**), ATF6α (**b**), and CHOP (**c**) in J774A.1 mouse macrophages pre-treated with increasing concentrations (2.5%, 5%, 10% *v*/*v*) of SE FAE or with SA for 24 h and subsequently stimulated or not with LPS. Results were obtained using the Western blot technique. Data are presented as mean ± SEM. Legend: SE FAE–*Sambucus ebulus* L. fruit aqueous extract; SA–100 μM salicylic acid; LPS–200 ng/mL lipopolysaccharides. * *p* < 0.05, ** *p* < 0.01 vs. untreated cells; # *p* < 0.05, ## *p* < 0.01 vs. LPS treatment.

Regarding the well-known relationship between inflammation and ER stress, we have analyzed intracellular protein levels of ER stress-related proteins: activating transcription factor 6 alpha (ATF6α), phosphorylated eukaryotic translation initiation factor 2 alpha (peIF2α), and their downstream target gene’s product C/EBP homologous protein (CHOP, growth arrest and DNA damage-inducible gene 153 (GADD153)) in a model of LPS-stimulated J744A.1 macrophages (Figure 5). Cells were pre-treated with increasing concentrations of 2.5%, 5% and 10% *v*/*v* (0.25 mg DW/mL, 0.5 mg DW/mL, 1 mg DW/mL respectively) SE FAE or SA for 24 h followed by LPS-stimulation for additional 24 h, and respective control treatments were performed as well.

#### 2.3.1. The Effect of LPS-Stimulation on ER Stress-Related Biomarkers in J774A.1 Macrophages

LPS treatment significantly induced the levels of peIF2α (Figure 5a), ATF6α (Figure 5b), and CHOP (Figure 5c) proteins by 38% (*p* < 0.05), 28% (*p* < 0.05), and 54% (*p* < 0.05), respectively.

#### 2.3.2. The Effect of SE FAE on ER Stress-Related Biomarkers in Non-Stimulated J744A.1 Macrophages

Applied alone, SE FAE in concentration of 2.5% *v*/*v* slightly increased peIF2α (by 16%, *p* < 0.05) (Figure 5a), but also slightly decreased the expression of ATF6α (by 11%, *p* < 0.05), similarly to the effect of SA (Figure 5b). The higher concentrations of the extract, 5% *v*/*v* and 10% *v*/*v*, reduced, in a dose-dependent manner CHOP levels by 20% (*p* < 0.05) and by 34% (*p* < 0.01), respectively (Figure 5c). The effect of SE FAE on CHOP levels was opposite to the effect of SA in non-stimulated with LPS macrophages. 2.3.3. The effect of SE FAE on ER stress-related biomarkers in LPS-stimulated J744A.1 macrophages.

#### 2.3.3. The Effect of SE FAE on ER Stress-Related Biomarkers in LPS-Stimulated J744A.1 Macrophages

Pre-treatment of macrophages with 10% v/v SE FAE significantly altered the LPS-stimulated levels of peIF2α (by 30%, *p* < 0.01), similarly to the effect of the SA (by 16%, *p* < 0.05). The same concentration of the extract similarly affected the expression of ATF6α (reduced by 27%, *p* < 0.05) (Figure 5b). Both, the 5% *v*/*v* and 10% *v*/*v* of the extract in the culture media significantly reduced in a dose-dependent manner the LPS-stimulated levels of CHOP by 19% (*p* < 0.05) and by 36%, respectively (Figure 5c). LPS-stimulated ATF6α and CHOP levels were not reduced by the pre-treatment with SA.

The original western blot gels presenting the changes in protein levels of iNOS, peIF2α, ATF6α and CHOP in J774A.1 mouse macrophages pre-treated with increasing concentrations (2.5%, 5%, 10% *v*/*v*) of SE FAE or with SA for 24 h and subsequently stimulated or not with LPS, are given in Figure S12.

#### 2.3.4. Correlation Analyzes of ER Stress-Related Biomarkers

**Figure 6:**
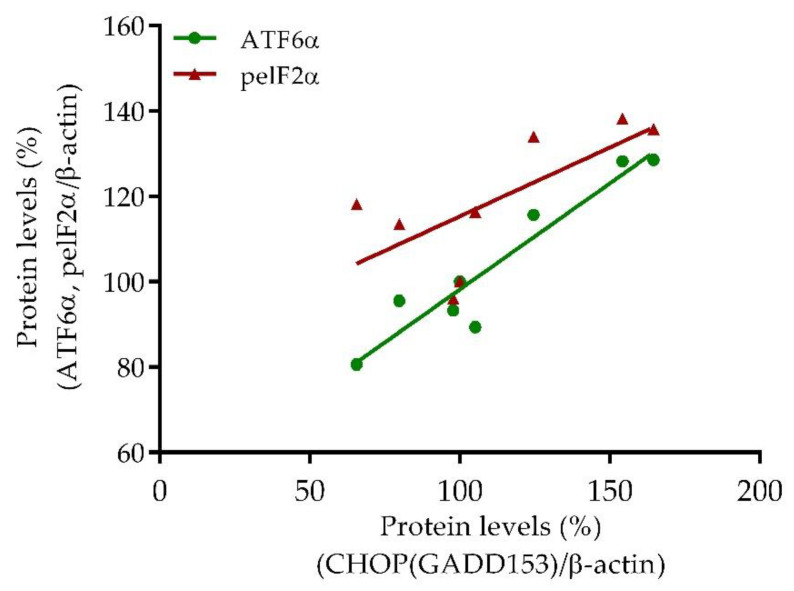
Correlations between protein levels of peIF2α and CHOP (red triangle) and of ATF6α and CHOP (green dot) in J774A.1 mouse macrophages pre-treated with increasing concentrations (2.5%, 5%, 10% *v*/*v*) of SE FAE and subsequently stimulated or not with LPS.

Based on the established clear and consistent dose-dependent effect of SE FAE in conditions of ± LPS stimulation, a subsequent correlation analyzes of transcription factors peIF2α and ATF6α and their downstream target CHOP were performed. High positive correlations between ATF6α and CHOP (r = 0.83, *p* < 0.05) and between peIF2α and CHOP (r = 0.67, *p* = 0.08) were established (Figure 6).

## 3. Discussion

### 3.1. Newly Detected Phytochemicals in SE Fruit Aqueous Extract

In the recent study we recognized as newly reported 10 AAs including 3 essential AAs, 8 OAs, 12 sugar acids and their phosphates, 16 alcohols, alcohol phosphates and their glycosides, 11 saccharides (mono-, di-, and tri-), 6 saturated and unsaturated fatty acids and 3 of their esters and 3 anthocyanins.

#### 3.1.1. Amino Acids

AAs are important for building cellular proteins, nucleotides, for maintaining acid-base balance and as neurotransmitters. Especially the essential AAs are valuable food components for living organisms. Other authors have reported three essential (Val, Leu and Thr) and three non-essential AAs (Ala, Gln and Tyr) in SE extracts [13]. In our study we have identified all of them except Ala. In addition, we have found three newly detected essential AAs (Ile, Phe and Lys) and six non-essential AAs (Pro, Gly, Ser, Asp, Asn, Gln) and Orn. In total, the content of AAs in the tested SE extract was 126.30 µg/mL, where the essential AAs comprise 30% of all AA content. Considering these findings, it could be concluded that SE fruits are a good source of both essential and non-essential AAs.

#### 3.1.2. Organic Acids

The total amount of all detected OAs was 110.66 µg/mL. We identified, for first time, seven OAs, including isocitric acid (16.37%) and succinic acid (11.42%), important substrates for the normal functioning of citric acid cycle and cell energy production. Fumaric and malic acid were previously reported in SE fruit extract [13]. Additionally, other authors reported the presence of citric acid in SE fruit extract, also important for energy metabolism [13]. Pyroglutamic acid (5-oxoproline) was found in the highest content (30.39% of all OA) in our samples. Pyroglutamic acid is very important as an intermediate in γ-glutamyl cycle involved in transmembrane amino acid transportation and for synthesis of the antioxidant glutathione. We may speculate that the high content of pyroglutamic acid in our samples may be due to a high amount of its keto derivative L-proline (15.84% of AAs) found in SE fruits [39].

#### 3.1.3. Sugar Acids and Alcohols

Regarding the content of sugar acids and sugar alcohols, the presence of pectic acid and sorbitol have been reported in the fruit of *S. nigra* [40,41]. Data in the literature concerning SE fruits content of sugar acids and sugar alcohols are not available.

In our study, the total amount of sugar alcohols was 268.62 µg/mL. For first time, we identified 17 sugar alcohols and their derivatives: sorbitol and its phosphate form (34.47% of sugar alcohols), glycerol, glycerol-3-phosphate and digalactosylglycerol (22.65%) and arabinitol (12.90%). Summarizing, the newly identified sugar alcohols and their derivatives comprise more than 68% of all quantified sugar alcohols in our samples. Therefore, SE fruits should be considered as a good natural source of sorbitol, glycerol and their derivatives.

The total amount of sugar acids in our samples was 115.49 µg/mL. For the first time, 12 sugar acids were identified; of them, the highest content was found for glucuronic acid (21.98%), galacturonic acid (16.28%), and glyceric acid (14.76%). Glucuronic and galacturonic acids are the most abundant sugar acids in the tested SE fruit extract, as they are the major components of plant polysaccharides, like cellulose and pectin.

#### 3.1.4. Saccharides

Literature data provide information mostly about glucose and sucrose content in SE fruit extracts [13]. We report new data regarding the saccharide content of SE fruit extract: the monosaccharides comprise 64.12% (263.61 µg/mL), followed by disaccharides 26.51% (108.98 µg/mL), and trisaccharides (mainly raffinose) 9.37% (38.52 µg/mL). Other identified monosaccharides were fructose, fructose-6-phosphate, arabinose, xylose, and mannose-6-phosphate. Disaccharides were presented by melibiose and trehalose as well.

#### 3.1.5. Fatty Acids and Fatty Esters

Data regarding the lipid composition of SE fruits are very limited. Most studies identified four sterols in SE fruit extract: brassicasterol, campesterol, stigmasterol, β-sitosterol [11,12]. In our samples, β-sitosterol (15.22 µg/mL) was the only sterol we detected. The newly identified fatty esters included 1-monopalmitin and monooctadecanoylglycerol. Other authors provide data regarding the presence of octadecanoic and octadecadienoic acids as well as palmitic, octadecenoic, dehydroabietic, oleic, oleanolic, ursolic, and maslinic acid [12]. We found the highest amount of any fatty acid for octadecadienoic acid (15.65 ±1.41 µg/mL) comprising 18.41% of all fatty acids (84.98 µg/mL), and five newly identified fatty acid (hexadecenoic, heptadecanoic, hexadecatrienoic, hexadecanoic and octadecatrienoic).

Oleanolic, ursolic, and maslinic acid are pentacyclic triterpenes known to possess anticancer properties [42,43]. Ursolic acid, particularly, reduces LPS-stimulated NFκB signaling [44], inflammatory cytokine production by inhibition JNK signaling [45], ER stress induced by high fat diet, and NFκB related inflammation [46]. It is assumed that ursolic acid may also improve insulin sensitivity [47,48]. In our previous study on healthy volunteers we reported that SE fruit tea intake improves lipid profiles, reducing total and LDL cholesterol serum levels and improving volunteers’ HDL/LDL ratios [22]. The intake of phytosterols including β-sitosterol may reduce total serum cholesterol [49] and low-density cholesterol [50]. As brassicasterol, campesterol [11] and β-sitosterol are among the phytosterols with known cholesterol-lowering activity [51,52,53], it is not surprisingly that SE fruit tea exerts a cholesterol-lowering effect.

#### 3.1.6. Phenolic Compounds

Compared with other species of the *Sambucus* genus, such as *S. nigra*, *S. cerulea*, and *S. racemose, S. ebulus* is the richest in total hydroxycinnamic acids, catechin, epicatechin and flavonols [15].

Anthocyanins are the predominant colored polyphenols in elderberries. In our study we identified three new anthocyanins: cyanidin-3-O-galactoside (the major anthocyanin in most plants), cyanidin-3-O-arabinoside and cyanidin-3-O-xyloside. In accordance with other studies, we also found in our samples cyanidin-3-O-glucoside, but not cyaniding-3-O-sambubioside [14]. Anthocyanins exerts numerous beneficial health effects including antioxidant, anti-inflammatory, anticancer, antidiabetic, anti-toxic, cardiovascular and nerve-protective capacities [54].

Flavanols, including catechin and epicatechin, were previously reported in SE fruits [15,16], as were proanthocyanidin dimers and trimers [55]. SE is the richest among the *Sambucus* sp. in catechins and epicatechins [15]. Epicatechin is the major proanthocyanidin monomer and a component of proanthocyanidin dimers and trimers. It is considered that one of the richest sources of proanthocyanidins are grape seeds [56]; proanthocyanidin dimer and trimer content in SE fruits is comparable to that in the grape seeds [55].

Resveratrol is the most abundant stilbene in plants. Grape peels are known as one of the best sources of resveratrol, containing on average 0.169 mg/g DW [57]. We found that *trans*-resveratrol-3-O-glucoside represents 5.19 mg/g DW. Thus, SE fruits and its FAE seems to be considerable sources of resveratrol. Resveratrol exerts a wide range of biological activities. It acts as calorie-restriction mimetic, increasing the levels of so-called survival protein SIRT1 and improves energy metabolism, decreases plasma glucose, triglycerides and inflammatory cytokines [58]. Its positive impacts on human heath are complemented by improved plasma antioxidant activity and reduced oxidative stress [59,60]. In obese individuals, resveratrol improves insulin sensitivity [61] and mitochondrial oxidative capacity when used in combination with epigallocatechin gallate [62].

Quinic acid is a compound conjugating with hydroxycinnamic acids to form their esters. Its presence in SE fruit tea (hot extraction) was reported previously by our group [55]. Recently, we also confirmed its availability in SE FAE (cold extraction).

Hydroxycinnamic acids are the most abundant phenolic acids in fruits, vegetables, and coffee beans [63]. They present as esters of hydroxycarboxylic acids, such as quinic acid or as glycosylated derivatives. Among them are caffeic acid, ferulic acid, chlorogenic acid, isoferulic acid and coumaric acid. There are data showing that SE fruits contain highest concentration of neochlorogenic acid and chlorogenic acid among all *Sambucus* sp., followed by *Sambucus cerulea* [15]. The same authors report also presence of p-coumaric acid-O-glucoside, 3-O-p-coumaroylquinic acid, and 4-O-p-coumaroylquinic acid in SE fruits. A study on SE fruit tea confirmed the presence of these hydroxycinnamic acids and those that we have also found in SE FAE [55]. There was no significant difference in hydroxycinnamic acid content between SE fruit tea prepared by hot and cold FAE extraction. In accordance with others, we have also found that the neochlorogenic acid followed by chlorogenic and 3-O-p-coumaroylquinic acid were the main hydroxycinnamic acids in SE FAE. The beneficial effects of hydroxycinnamic acids as potential chemo-preventives are associated to their antioxidant activity [64]. Coumaric and ferulic acid and their amides significantly reduce LPS-stimulated NO synthesis, iNOS protein content and mRNA levels in RAW 264.7 macrophages, thus presuming a mechanisms of their anti-inflammatory activity [65]. Plant extracts rich in neochlorogenic acid possess various biological activities, including antioxidant and anti-inflammatory [66,67,68]. As SE FAE is rich in hydroxycinnamic acids and their derivatives, it could be suggested that hydroxycinnamic acids are the main bioactive components determining its antioxidant, and anti-inflammatory.

The most abundant flavonol glycoside in *Sambucus* sp. is quercetin-3-O-rutinoside (rutin) [15]. Other flavonol glycosides detected in SE include quercetin glycosides, followed by kaempferol glycosides, and isorhamnetin glycosides [12,15,55]. In our samples the total amount of flavonols was 195.35 µg/mL, comprising only 3.35% of all analyzed polyphenols. In addition, the quercetin glycosides (128.63 µg/mL) dominate over kaempferol glycosides (66.72 µg/mL), representing 65.85% and 34.15% respectively of all identified flavonols in SE FAE. The presence of flavonols, quercetin and kaempferol in SE fruit extracts was widely reported in the literature [16]. Other studies provide data regarding the content of rutin [9,16,55], isoquercetin and hyperoside [9,15,55], kaempferol 3-O-rutinoside [15], isorhamnetin-3-O-laminaribioside [12], isorhamnetin 3-O-rutinoside (narcissin) [12,15], isorhamnetin 3-O-glucoside [9,12], and myricetin [16] in SE fruit extracts. In accordance with the data of others, we have also identified quercetin-3-O-rhamnosyl-galactoside, quercetin-3-O-rhamnosyl-glucoside, guaiaverin, quercetin-3-O-xyloside, kaempferol-3-O-galactoside, astragalin, kaempferol-3-O-rhamnosyl-galactoside, kaempferol-3-O-rhamnosyl-glucoside, kaempferol-3-O-arabinoside, kaempferol-3-O-xyloside in our samples [55]. Flavonoid-rich herb extracts possess strong antioxidant and anti-inflammatory activities [69,70]. Both isoquercetin and hyperoside exert antioxidant and anti-inflammatory [71,72] effect. Similarly, quercetin and rutin exhibit anti-inflammatory, anti-cancer, anti-bacterial and anticonvulsant activities [73,74,75].

### 3.2. SE FAE Modulates mRNA and Protein Levels of Inflammation-Related Biomarkers in LPS-Challenged J774A.1 Macrophages

The anti-inflammatory effect of polyphenols is due to the decreased activation of macrophages and T-lymphocytes and the suppressed production of cytokines and chemokines or their receptors. Polyphenols such as resveratrol, catechin and quercetin, found in SE fruits, inhibit NFκB-dependent production of ICAM and VCAM in endothelial cells, as well as the expression of MCP-1 receptors CCR1 and CCR2 [76,77]. Inhibition of the latter reduces the chemotaxis of leukocytes to the site of inflammation and the subsequent increased production of IL-6. Anthocyanin metabolites reduce TNFα-induced expression of MCP-1 and ICAM, and thus combat oxidative stress. In models of LPS-induced inflammatory response of macrophages, anthocyanidin- and anthocyanin-rich extracts inhibit iNOS transcription and iNOS and COX-2 translation by targeting the NFκB and MAPK kinase signaling pathways [78,79]. Karlsen et al. [80] reported that blackcurrant and blackberry polyphenols significantly inhibited NFκB in LPS challanged monocytes isolated from healthy adults.

In our previous study we found that SE FAE reduces LPS activated mRNA expression of NFκB, which correlated with decreased transcription levels of glutamate–cysteine ligase and glutathione peroxidase enzymes [18]. Neochlorogenic and chlorogenic acid, also found in SE FAE, suppress LPS-stimulated activation of NFκB patway resulting in reduced iNOS synthesis and activation of COX-2, thus decreasing NO, prostaglandin E2, TNFα, IL-1β, and IL-6 levels in RAW 267 macrophages [81]. Neochlorogenic and chlorogenic acid-rich plant extracts significantly reduce the carrageenan-induced paw edema in rats, in vivo [81]. Coumaric and ferulic acids were found to reduce LPS-stimulated iNOS protein and mRNA levels [65]. Few studies have reported the strong antioxidant, anti-inflammatory and antidiabetic properties of cyanidin-3-O-galactoside, one of the main anthocyanin in SE FAE [54]. Ursolic acid, found in SE leaves, reveals anti-inflammatory activity by reducing TNFα-induced expression of ICAM-1 and VCAM-1 in human umbilical vein endothelial cells [82]. Ursolic acid reduces LPS-stimulated NFκB [44] and JNK signaling, thus inhibiting inflammatory cytokine production [45]. It was reported, also, that it may combat ER stress- and NFκB-related inflammation in animals on a high-fat diet [46]. We also found an anti-inflammatory effect of SE FAE, which may be due to the presence of ursolic acid in SE fruits reported by others [12].

LPS stimulates the gene expression of cytokines IL-1β, TNFα and IL-6, chemokine ICAM-1 and the enzymes COX-2 and iNOS by activating the NFκB-dependent signaling pathway [83,84,85,86,87,88]. The activation of iNOS results in increased production of ONOO¯ and further stimulation of COX2 gene expression and prostaglandin E2 production [89]. Recently we observed that the pre-treatment with SE FAE significantly reduces LPS-stimulated transcription of pro-inflammatory cytokines IL-1β, TNFα, IL-6, the chemokines MCP-1, ICAM-1, enzymes COX-2, iNOS, as well as the protein levels of iNOS.

The effect was comparable to that of salicylic acid, a known anti-inflammatory agent, used in our study as a positive control. The possible mechanism behind the observed anti-inflammatory effect of SE extract might due to the presence of neochlorogenic acid, chlorogenicacid, ursolic acid, resveratrol, catechin and quercetin, by suppressing the NFκB signaling pathway. Additional mechanism for reducing COX2 activity and prostaglandin production might be the direct NO^.^ radical-scavenging activity of SE FAE [8].

Moreover, when applied alone, the lower doses of SE FAE induce the transcription of IL-6, TNFα and MCP-1 by two-fold; COX2 and iNOS transcription by three-fold and iNOS protein expression (*p* < 0.05). These results support the traditional application of dwarf elderberries in folk medicine as an effective immunostimulant. Our previous study reported increased NFκB, glutamate–cysteine ligase and glutathione peroxidase transcription and thus confirms the immunostimulatory effect of SE FAE [18]. Immunostimulatory effect was also proven for *S. nigra,* another member of the genus *Sambucus* [90].

NADPH oxidase (NOX), is one of the major enzymes in vascular endothelial cells, catalyzing the formation of a superoxide radical anion [91]. Endothelial eNOS, as well as iNOS, produce NO, which reacts with a superoxide radical anion forming highly reactive ONOO¯ [92,93] and contributing to the development of oxidative stress. NOX is highly active in activated macrophages, taking part in a respiratory burst for destroying bacterial cell walls [94]. NOX is among the newly established target molecules in the treatment of hypertension and atherosclerosis, and concomitant pathologies such as diabetes and cardiovascular diseases [91,95].

By suppressing the LPS-induced gene expression of NOX subunit Noxo1, SE FAE exhibits strong antioxidant and anti-inflammatory activity. The effect of the extract on LPS-induced Noxo1 transcription was similar to that of SA. Both SE FAE and SA are very effective in completely neutralizing LPS-induced Noxo1 overexpression. Compounds such as epigallocatechin gallate, quercetin and isorhamnetin, the derivatives of which are found in SE fruits, were shown to target Noxo1 [96,97,98,99,100,101], while resveratrol decreases NOX activity [102]. NOX, and in particular its Noxo1 subunit, has been suggested as playing an important role in the IL-1β-dependent activation of NF-κB [103]. Therefore, the inhibition of Noxo1 gene expression is one potential mechanism by which SE fruits suppress the NFκB-dependent expression of pro-inflammatory genes and proteins such as iNOS.

FABP4 one of the fatty acid-binding proteins is expressed in both adipocytes and macrophages [104,105]. Macrophages are specific target cells for Fabp4 and its deficiency prevents atherosclerosis [100]. High FFA contribute to the development of atherosclerosis, are proinflammatory and activate TLR4 signaling cascades. The same signaling pathway is also activated by LPS [25]. In animal models of obesity and insulin resistance it was shown that the inhibition of FABP4 protein in macrophages reduces inflammatory cytokines (MCP-1, IL-1β, IL-6 and TNFα) and the formation of atherosclerotic lesions and foam cells and improves insulin sensitivity [106,107]. Recently, we studied the effect of SE FAE on the transcription levels of the Fabp4 gene. SE FAE, applied alone, slightly stimulated Fabp4, as was observed for other studied inflammatory genes. On the other hand, in LPS-stimulated cells, pre-treatment with the same concentrations of SE FAE completely prevents LPS-stimulated Fabp4 transcription. These findings suggest another possible anti-inflammatory mechanism of SE FAE action.

SIRT-1 is among the most studied sirtuins from class III histone deacetylases, which activation improves obesity related insulin resistance [108] and possesses anti-inflammatory potential [25]. Sirt-1 activators, such as resveratrol, may inhibit ICAM1 and TNFα induction [109]. In our study, SE FAE induces the expression of Sirt-1. The same result is observed in macrophages pre-treated with the extract and stimulated by LPS. SIRT-1 decreases serine phosphorylation in IRS-1, improves insulin signaling and, as a consequence, increases glucose transport [110]. The same mechanism is involved in improving insulin sensitivity decreased by TNF-alpha. These findings suggest another possible anti-inflammatory and insulin sensing mechanism of SE FAE, although these mechanisms are unclear and require additional studies in future.

### 3.3. SE FAE Modulates Levels of ER Stress-Related Proteins in LPS-Challenged J774A.1 Macrophages

Activation of ER stress may lead to the phosphorylation of JNK and IKK, which is well-known to promote NFκB signaling and the consequent inflammation [32] accompanied by JNK-mediated phosphorylation of IRS 1/2 [111] to impair insulin signaling. On the other hand, triggered by viral or bacterial infections, the production of TNFα, IL-6, IL-1β, and INFγ may amplify the ER stress in many cell types including macrophages, pancreatic β cells and hepatocytes [112,113]. Since both the processes of inflammation and ER stress may result from each other, we analyzed the expression of three important ER stress-related proteins as potential mechanisms to explain the observed anti-inflammatory potential of SE FAE.

We have observed a significant increase in protein levels of transcription factors ATF6α and peIF2α and their downstream target CHOP in LPS-stimulated macrophages. Applied alone SE FAE downregulated the synthesis of CHOP at a dose-dependent manner and slightly that of ATF6α. SE FAE significantly reduced LPS-stimulated CHOP levels. The decrease in ATF6α levels and the phosphorylated eIF2α in LPS-stimulated macrophages provides evidence for a possible mechanism by which the extract inhibits CHOP synthesis. This cytoprotective mechanism in the case of stimulated ER stress is confirmed by the established high significant correlation between protein levels of CHOP and transcription factors (Figure 5). This may explain the previously reported cytoprotective effects of SE fruits as well [9,19]. SA reduced only the LPS-stimulated peIF2α protein levels. It should be noted that the SE FAE effect was in a similar direction, however it was stronger than that of SA. With regard to the well-known anti-inflammatory activities of SA [114] and the links between inflammation and the activation of ER stress, we expected that SA might have reducing effect on ER stress-related biomarkers. However, SA did not exhibit any protective effect against LPS-stimulated ATF6α and CHOP levels, as SE FAE, in contrast, did. According to this observation, we may suggest that the SE FAE uses mechanisms different from those of SA, resulting not only in reduced transcription of inflammatory markers but also in the translation of ER stress-related ones.

ER stress could be activated by high levels of FFAs, similarly to inflammation, excess nutrients, improperly folded proteins and local hypoxia, which is characteristic of obesity. This results in increased oxidative stress in the liver and in adipose tissue of obese animals [115]. An interesting fact is the established activated expression of Fabp4 in macrophages and its association with the development of ER stress and inflammation [106]. In accordance with previous analyses, the suppression of Fabp4 in macrophages protects cells from the FFA-induced inflammatory process, which may result in increased insulin sensitivity and glucose tolerance [106]. The ability of the SE FAE to inhibit LPS-induced transcription of Fabp4 suggests that it would also have a protective effect in combating ER stress. FFA and glucose activate PERK-mediated phosphorylation and activation of eIF2α and RNA splicing of Xbp-1 in obese rat and human adipocytes [116,117]. CHOP is induced predominantly by the PERK/eIF2α/ATF4 signaling cascade associated with ER stress, as well as by the IRE1α/Xbp-1 signaling pathway and the ATF6α transcription factor in different pathological conditions, including diabetes [118,119,120,121].

The induction of CHOP is associated with the activation of apoptosis and DNA damage. Its induction in humans and animal macrophages is associated with the detachment of atherosclerotic plaques in atherosclerosis [122]. The production of superoxide by NOX in atherosclerotic plaque-associated macrophages activates CHOP and subsequent ER stress-mediated cell death [123]. ER stress may stimulate NFκB, by Ca^2+^- and reactive oxygen species-dependent mechanisms [124] and the activation of PERK/eIF2α-mediated phosphorilation of IKK [125]. Another important mediator of the ER stress-related activation of NFκB signaling and the consequent TNFα, IL-6 and IL-1β cytokines production is iNOS [34]. This transforms iNOS enzyme into a cross point of inflammation and ER stress, and, consequently intoa possible therapeutic targets.

By preventing the LPS-induced transcription of iNOS and Noxo1 and the subsequent translation of iNOS protein, SE FAE may reduce superoxide radical and ONOO^-^ production, thus reducing the activation of ER stress-related inflammation; whereas, suppressing CHOP synthesis by suppression of peIF2 and ATF6α possess another important mechanism for combating ER stress-related activation of inflammation and cytokine production. These are the first results confirming that SE, and in particular SE fruits, could suppress the induction of inflammation by suppressing the activation of ER stress.

The complex phytochemical composition of SE FAE presumes that it acts at different levels on inflammation and ER stress regulatory cascades. SE FAE acts at different cross-points starting from the direct scavenging of reactive oxygen species, through the regulation of gene transcription, to protein synthesis.

Our results contribute to the relationship between inflammation, ER stress and insulin resistance. We may speculate that SE FAE could have a beneficial effect in preventing atherosclerosis, insulin resistance and diabetes type 2, but this requires additional specific studies to confirm the possible insulin sensing effect of the SE fruits.

## 4. Materials and Methods

### 4.1. Plant Material

Well-ripened fruits of *Sambucus ebulus* L. were harvested from North-Eastern Bulgaria in the period August–September, 2014 and were dried in the dark at room temperature. SE FAE was prepared using 150 mg finely grounded dry fruits, extracted three times with 3 mL distilled water for 3 min in a vortex mixer (2000 rpm), at room temperature. After centrifugation (5 min, 3500 rpm) the supernatants were collected and diluted up to 15 mL with PBS buffer (pH = 7.4) for cell-culture experiments or with distilled water for phytochemical analyses. A specimen from *S. ebulus* L. fruits was deposited under No. 108144 in the Herbarium SO (by Index Herbariorum) of Sofia University St. Kliment Ohridski, Faculty of Biology.

### 4.2. Phytochemical Analysis

#### 4.2.1. Extraction

The samples were filtered through a 0.45-µm PTFE filter (Waters, USA) and the filtrates were loaded onto a reverse phase solid phase extraction column (Discovery^®^ DSC-18, 5 g, 20 mL) (Sigma-Aldrich Co. LLC, St. Louis, MO, USA). The SPE columns were activated rinsed with 40.0 mL distilled water and 1 mL of the filtered sample was loaded onto the SPE column. Three fractions were isolated: anthocyanin fraction (C)—eluted with 2 × 12 mL 0.1% (*v*/*v*) formic acid in acetonitrile; non-anthocyanin fraction (B) containing phenolic acids, flavonols, flavonols, eluted with a 2 × 12-mL ethyl acetate; polar fraction (A), containing organic acids, aminoacids and carbohydrates-eluted with 2 × 12-mL 0.2% (*v*/*v*) formic acid in water. The eluates were evaporated to dryness under reduced pressure at a temperature below 40 °C.

#### 4.2.2. Analysis of Polar Fraction (A)

An aliquot (0.2 mL) of fraction A was submitted to lyophilisation for 6 h at −20 °C. The dry residue was subjected to the following derivatization protocol: 300.0 µL solution of methoxyamine hydrochloride (20.0 mg/mL in pyridine) was added to a residue and the mixture was heated on Thermo-Shaker TS-100 (1 h/70 °C/300 rpm). After cooling, 100.0 µL N,O-Bis (trimethylsilyl)trifluoroacetamide (BSTFA) were added to the mixture, then heated on Thermoshaker, Analytik Jena AG, Jena, Germany (40 min/70 °C/300 rpm). Then, 1.0 µL of the solution was injected in the GC–MS system (Agilent GC 7890, Agilent MD 5975). The separations were done on a chromatographic column HP-5ms (length 30 m, diameter 0.32 mm, film thickness 0.25 μm) at a gradient temperature mode: initial 100 °C for 2 min; ramp up to 180 °C with 15 °C/min for 1 min; ramp up to 300 °C with 5 °C/min for 10 min. Injector and detector temperatures were set at 250 °C; the velocity of the carrier gas helium was set at 1.0 mL/min. The MS scanning was in the range 50–550 m/z.

#### 4.2.3. Analysis of Fractions B and C

Fractions B and C were analyzed using LC-PDA-ESI-MS/MS chromatographic system; in negative ESI mode for fraction B, and in positive ESI mode for fraction C as previously described [55].

For the analysis of polyphenolics, the dry residues of fraction B and C were dissolved in 200 μL metahnol:formic acid, (99:1 *v*/*v*), the solution was filtered through a 0.22 µm PTFE filter and 2 µL of the filtrate were injected into LC-PDA-ESI-MS/MS system.

An LTQ Orbitrap mass spectrometer (Thermo Scientific, Hemel Hempstead, UK) equipped with an ESI source (in negative mode) was used for accurate mass measurements. Operation parameters were as follows: source voltage—4 kV; sheath, auxiliary and sweep gas −20, 10 and 2 arbitrary units, respectively; capillary temperature was275 °C. The samples were analyzed in full-scan mode at a resolution of 30,000 at m/z 400 and data-dependent MS/MS events were acquired at a resolving power of 15,000. The most intense ions were detected during full-scan MS-activated data-dependent scanning. Ions that were insufficiently intense were analyzed in MS2 mode with a resolution power of 15,000 at m/z 400. An isolation width of 100 amu was used. Precursors were fragmented by a collision-induced dissociation with energy of 30 V and an activation time of 10 ms. The mass range in FTMS mode was from m/z 100 to 1000. The data analyses were performed using XCalibur software v2.0.7 (Thermo Fisher Scientific, Hemel Hempstead, UK).

Chromatographic separations were performed on an Accela chromatograph (Thermo Scientific, Waltham, MA, USA) equipped with a quaternary pump, a photodiode array detector (PDA) and a thermostated autosampler. A Kinetex C18 column (100 Å, 2.6 μm, 150 × 2.1 mm) was used to perform chromatographic separations (Phenomenex Inc., Torrance, CA, USA). The elution was done in a gradient mode with water/0.1% formic acid (solvent A) and acetonitrile (solvent B) at a constant flow rate of 0.3 ml/min. The gradient composition of the mobile phase was as follows: 0 min, 10% B; 1 min, 10% B; 15 min, 30% B; 22 min, 50% B; 28 min, 100% B; 34 min, 100% B, 36 min, 10% B. prior each analysis the column was equilibrated for 6 min. The total run time was 36 min.

#### 4.2.4. Identification and Quantitative Analysis

The identification of compounds in fraction A was done either by comparison the retention times and Kovats indexes (RI) of the tested compounds with the same parameters of the corresponding pure standards or with mass spectra from the Golm Metabolome Database (http://csbdb.mpimp-golm.mpg.de/csbdb/gmd/gmd.html, 30 August 2021) and NIST’08 (National Institute of Standards and Technology, Gaithersburg, MD, USA) libraries. 

The quantification of phenolics in fractions B and C was performed by the external standard method as previously described [55]. Fifteen phenolic compounds were confirmed by comparing their retention times, exact masses and fragmentation patterns with corresponding standards. The identification of the remaining compounds without available standards was based on accurate mass measurements of the [M − H]^−^ ions and the fragmentation patterns, which was compared with the literature data.

### 4.3. Cell Culture

J774A.1 mouse macrophages were purchased from American Type Culture Collection (ATCC, Manassas, VA, USA). Cells were cultured in 75 cm^3^ flasks at 37 °C in a humidified chamber (CO2CELL48, MMM Medcenter Einrichtungen GmbH, Planegg, Germany) with 95% air and 5% CO2 in Dulbecco’s Modified Eagle Medium (DMEM, with 4.5 g/L of glucose and L-glutamine) (LONZA, Verviers, Belgium) supplemented with 10% heat-inactivated fetal bovine serum (FBS, Sigma-Aldrich, Taufkirchen, Germany) and 1% antibiotics (100 U/mL penicillin, 100 µg/mL streptomycin) (LONZA, Verviers, Belgium). Cells were sub-cultivated until they reach 80% confluence. Cell counts were prepared in quadruplicate by 0.4% trypan blue exclusion dye (Chemapol, Prague, Czech Republic) using a counting Burker chamber.

### 4.4. Study Design

The experimental model involved macrophage cells seeded in 6-well plates (2 × 10^5^ cells/well) and allowed to adhere overnight. The study design included the following experimental groups: (1) cells treated only with LPS; (2) cells treated with SE FAE; (3) cells pre-treated with SE FAE and consequently challenged with LPS. For control groups, we used untreated cells (blank); salicylic acid-treated cells (positive, anti-inflammatory control) and cells pre-treated with salicylic acid and consequently challenged with LPS.

Cells were pre-treated with SE FAE with increasing concentrations of 2.5%, 5% and 10% *v*/*v* (0.25 mg DW/mL, 0.5 mg DW/mL, 1 mg DW/mL, respectively) or salicylic acid (100 μM) (Merck, Germany) dissolved in DMEM (with 4.5 g/L glucose, w/o phenol red and L-glutamine) supplemented with 10% heat-inactivated FBS, 100 U/mL penicillin/100 µg/mL streptomycin mixture and 2 mM L-glutamine. After 24 h cells were treated with 200 ng/mL LPS (*Escherichia coli* 026:B6, Sigma-Aldrich, Taufkirchen, Germany) or not, by the simple refreshing of culture media and incubated for additional 24 h. Following the last incubation period, the cells were lysed and total RNA or total protein were extracted and subjected to subsequent analyses. All treatments were performed in triplicate.

### 4.5. Gene Expression Analysis

#### 4.5.1. RNA Extraction and cDNA Synthesis

Total RNA was extracted using TRI reagent (Ambion, Waltham, MA, USA) according to the manufacturers’ requirement. RevertAid First Strand cDNA Synthesis kit (ThermoFisher Scientific, Waltham, MA, USA) was used to reversely transcribe 20 ng of total RNA using oligo (dT)_18_ priming strategy. Following the manufacturers’ protocol reaction conditions in final volumes of 10 µL were provided. cDNA synthesis was performed on GeneAmp PCR 7500 thermal cycler (Applied Biosystems, Waltham, MA, USA). After synthesis cDNA was diluted by adding of 30 µL nuclease-free distilled water to each sample and stored at −80 °C.

#### 4.5.2. qPCR Analysis

Gene transcription levels were analyzed using the qPCR method and performed on an ABI PRISM 7500 (Applied Biosystems, Waltham, MA, USA). KAPA SYBR^®®^ FAST qPCR Master Mix (2X) with low ROX (KAPA Biosystems, Cape Town, South Africa) was used. The amplification reaction’s final volume was 5 µL in 96-well plates, with 0.39 µL of cDNA template. Final concentration of primers’ was 300 nM. Reaction conditions were as follows: 95 °C/5 min; 40 cycles at 95 °C/15 sec and 60 °C/1 min. A dissociation step was added to the instrument’s protocol to check for nonspecific amplification. As an internal control, the β-actin gene was used. Relative gene expression levels were calculated using the 2^−ΔΔCt^ method [126]. The used primer sequences (Sigma-Aldrich, Taufkirchen, Germany) for each gene analyzed are presented in Table 3. Expression levels of mRNA are presented as relative units (RU) compared to the untreated control group of cells, where the levels of mRNA expression were considered to be equal to 1. Analyses were performed in triplicate and included all cell groups in one plate while testing the expression of each gene.

### 4.6. Protein Expression Analysis

#### 4.6.1. Protein Extraction and Quantification

Total protein was extracted using Pierce™ IP Lysis Buffer (25 mM Tris-HCl pH 7.4, 150 mM NaCl, 1 mM EDTA, 1% NP-40 and 5% glycerol) (Thermo Fisher Scientific, Waltham, MA, USA) with freshly added Halt™ Protease and Phosphatase Inhibitor Cocktail (Thermo Fisher Scientific, Waltham, MA, USA), according to the manufacturers’ requirement. Prior to our analyses, total protein concentration was measured using a Bradford reagent (Protein assay dye concentrate, Bio-Rad Laboratories; Hercules, CA, USA) and calculated against a standard curve of standard bovine serum albumin (BSA) (Thermo Fisher Scientific, Waltham, MA, USA) dilutions.

#### 4.6.2. Western Blotting

Protein lysates were subjected to SDS-PAGE, electrotransferred to a polyvinylidene difluoride membranes (PVDF; Merck Millipore; Billerica, MA, USA) and subsequently incubated with the following antibodies: ATF6α (90 kDa) (1:1000, sc-166659), CHOP (GADD153) (26 kDa) (1:1000, sc-7351), peIF2α (Ser52) (36 kDa) (1:1000, sc-12412), iNOS (1300 kDa) (1:1000, sc-7271) (Santa Cruz Biotechnology; Santa Cruz, CA, USA) and β-actin (42 kDa) (1:5000; A5316) (Sigma; St. Louis, MO, USA) after incubating the membranes with 3% BSA (β-actin, ATF6α), 5% BSA (peIF2α) or 5% skim milk (CHOP, iNOS) blocking buffer. Specific antigen–antibody bindings were detected using horseradish-peroxidase conjugated secondary antibodies (Dako Denmark; Glostrup, Denmark) and an enhanced chemiluminescence detection method, according to the manufacturer’s instructions (Pierce ECL Western Blotting Substrate; Thermo Scientific, Waltham, MA, USA) as described previously [127,128]. Autoradiographic films (Fujifilm; Tokyo, Japan) were scanned and the band’s signal was quantified by densitometry using ImageJ-1.53 software (National Institutes of Health, Bethesda, MD, USA). Values were expressed relative to β-actin.

### 4.7. Statistical Analysis

GraphPad Prism v7.0 software (GraphPad Software, Inc.; La Jolla, CA, USA) was used to perform the statistical analyses (Student’s *t*-tests, Spearman correlation, 95% CI). The values of *p* < 0.05 were considered as significant. Data were presented as mean ± SD (concentration of phytochemical) or ± SEM (mRNA and protein expression levels). All analyses and treatments were performed in triplicates.

## 5. Conclusions

The SE FAE is confirmed to be rich in phytochemicals, predominantly hydroxycinnamic acids, anthocyanins, proanthocyanidins and resveratrol, with strong antioxidant-, anti-inflammatory- and ER stress-reducing potential, as well as in AAs including essential ones, organic acids, alcohols and saturated and unsaturated fatty acids and esters, some of them reported for first time in SE fruits.

Considering the results, we may conclude that SE FAE, applied alone, possesses immunostimulating potential, without promoting any additional stress, such as ER stress. It may reduce the ER stress-related expression of the CHOP protein, independently from its immunostimulating potential. The SE fruit extract exerted significant antioxidant and anti-inflammatory action, decreasing the LPS-stimulated transcription of oxidative stress, inflammation, atherosclerosis and insulin resistance-related cytokines, chemokines and enzymes, as well as the translation of iNOS. The herb extract possesses significant ER stress-reducing potential, by suppressing the LPS-stimulated synthesis of peIF2α, ATF6α and CHOP proteins. Taken together, these results reveal a new possible mechanism explaining the anti-inflammatory potential of SE fruits, by targeting ER stress and related biomarkers.

These findings are in concordance with the traditional usage of SE fruits and its FAE as potential natural immunomodulation preparation, beneficial in the prevention or treatment of oxidative stress- and inflammation-related conditions.

## Figures and Tables

**Table 1:** List of polar phytochemicals identified in the analyzed polar fraction (A) of SE FAE using GC-MS technique. The concentration was given in µg/mL extract. Results are presented as mean ± standard deviation.

Compound	Content, µg/mL
**Amino Acids**	
*L-Valine*	3.02 ± 0.21
*L-Leucine*	8.06 ± 0.56
*L-Isoleucine*	8.48 ± 0.59
*L-Proline*	20.01 ± 1.40
*L-Threonine*	3.89 ± 0.27
*L-Phenylalanine*	10.25 ± 0.72
*L-Lysine*	4.37 ± 0.31
Glycine	3.78 ± 0.26
Serine	2.59 ± 0.18
L-Aspartic acid	16.32 ± 1.14
L-Asparagine	6.19 ± 0.43
L-Glutamic acid	1.34 ± 0.09
L-Glutamine	22.99 ± 1.61
DL-Ornithine	12.36 ± 0.86
L-Tyrosine	2.66 ± 0.19
**Organic Acids**	
Succinic acid	12.64 ± 0.88
Fumaric acid	6.61 ± 0.46
Malic acid	9.22 ± 0.65
Pyroglutamic acid (5-oxoproline)	33.63 ± 2.35
4-Aminobutyric acid	5.69 ± 0.40
2-Hydroxyglutaric acid	4.07 ± 0.29
2-Ketoglutaric acid	8.02 ± 0.56
Phenylpyruvic acid	2.18 ± 0.15
2,3-Dihydroxybutanedioic acid	10.49 ± 0.73
Isocitric acid	18.12 ± 1.27
**Sugar Acids and Alcohols**	
Glycerol	36.12 ± 2.53
Digalactosylglycerol	6.99 ± 0.63
Glyceric acid	17.05 ± 1.19
Threitol	7.66 ± 0.54
Erythreol	2.09 ± 0.15
Erithreonic acid	2.65 ± 0.19
Threonic acid	8.40 ± 0.59
Xylitol	4.20 ± 0.29
Arabinitol	34.65 ± 2.43
Pentonic acid	7.69 ± 0.54
L-Glycerol-3-phosphate	17.72 ± 1.24
Ribonic acid	4.76 ± 0.33
Manitol	2.98 ± 0.21
Sorbitol	49.26 ± 3.45
Glucuronic acid isomer	8.49 ± 0.59
Galactitol	1.91 ± 0.13
Galacturonic acid isomer	15.91 ± 1.11
Glucuronic acid isomer	13.03 ± 0.91
Gluconic acid isomer	1.78 ± 0.12
Galacturonic acid isomer	2.89 ± 0.20
Glucuronic acid isomer	3.87 ± 0.27
Galactonic acid	6.33 ± 0.44
Gluconic acid isomer	3.71 ± 0.26
Glucaric acid	14.00 ± 0.98
Galactaric acid	3.38 ± 0.24
Myo-inositol	6.71 ± 0.47
Galactosylglycerol	22.50 ± 1.58
Sorbitol-6-phosphate	43.32 ± 3.03
myo-Inositol-1-phosphate isomer	5.64 ± 0.39
myo-Inositol-2-phosphate isomer	7.43 ± 0.52
Gluconic acid-6-phosphate	1.54 ± 0.11
myo-Inositol-1-phosphate isomer	3.30 ± 0.23
myo-Inositol-2-phosphate isomer	6.87 ± 0.48
Maltitol; alpha-D-Glc-(1,4)-D-sorbitol	4.90 ± 0.34
Galactinol isomer; alpha-D-Gal-(1,3)-myo-Inositol	0.69 ± 0.05
Galactinol isomer; alpha-D-Gal-(1,3)-myo-Inositol	3.67 ± 0.26
**Saccharides (mono-, di-, and tri-)**	
Xylose methoxyamine	5.94 ± 0.42
Arabinose methoxyamine	12.65 ± 0.89
Fructose isomer	14.31 ± 1.00
Fructose isomer	18.89 ± 1.32
Sorbose isomer	28.11 ± 1.97
Sorbose isomer	21.35 ± 1.49
Galactose isomer	35.19 ± 2.46
Galactose isomer	13.86 ± 0.97
Glucose isomer	17.34 ± 1.21
Glucose isomer	13.59 ± 0.95
Fructose-6-phosphate isomer	16.20 ± 1.13
Mannose-6-phosphate isomer	3.47 ± 0.24
Galactose-6-phosphate isomer	18.79 ± 1.32
Glucose-6-phosphate isomer	30.27 ± 2.12
Fructose-6-phosphate isomer	5.81 ± 0.41
Galactose-6-phosphate isomer	3.32 ± 0.23
Glucose-6-phosphate isomer	4.52 ± 0.32
Sucrose; alpha-D-Glc-(1,2)-beta-D-Fru isomer	24.81 ± 1.74
Trehalose; alpha-D-Glc-(1,1)-alpha-D-Glc isomer	10.10 ± 0.71
Melibiose isomer; alpha-D-Gal-(1,6)-D-Glc isomer	18.59 ± 1.30
Melibiose isomer; alpha-D-Gal-(1,6)-D-Glc isomer	18.80 ± 1.32
Sucrose; alpha-D-Glc-(1,2)-beta-D-Fru isomer	20.55 ± 1.44
Trehalose; alpha-D-Glc-(1,1)-alpha-D-Glc isomer	16.13 ± 1.13
Raffinose; alpha-D-Gal-(1,6)-alpha-D-Glc-(1,2)-beta-D-Fru isomer	12.91 ± 0.90
Raffinose; alpha-D-Gal-(1,6)-alpha-D-Glc-(1,2)-beta-D-Fru isomer	25.61 ± 1.79
**Saturated, unsaturated acids and esters**	
9-(E)-Hexadecenoic acid	8.52 ± 0.77
9-(Z)-Hexadecenoic acid	6.57 ± 0.59
Heptadecanoic acid	7.56 ± 0.68
Hexadecatrienoic acid	4.85 ± 0.44
Hexadecanoic acid (Palmitic acid)	6.56 ± 0.59
Heptadecanoic acid	6.06 ± 0.55
9,12-(Z,Z)-Octadecadienoic acid (Linoleic acid)	9.69 ± 0.87
9,12,15-(Z,Z,Z)-Octadecatrienoic acid (Linolenic acid)	8.42 ± 0.76
Octadecanoic acid (Stearic acid)	11.12 ± 1.00
(2E,4E)-2,4-Octadecadienoic acid	15.65 ± 1.41
1-Monopalmitin	13.80 ± 1.24
Monooctadecanoylglycerol	8.62 ± 0.78
beta-Sitosterol	15.22 ± 1.37

All metabolites are trimethylsilyl derivatives; SE FAE–*Sambucus ebulus* L. fruit aqueous extract; essential AAs are given in *italic*. Additional data regarding chromatographic parameters and total ion chromatogram of tested polar compounds are given in Table S1 and Figure S1, respectively.

**Table 2:** Polyphenolic compounds identified in non-anthocyanin fraction (B) and anthocyanin fraction (C) of the SE FAE using LC-PDA-ESI-MS/MS technique. The concentrations are given in µg/mL extract. Results are presented as mean ± standard deviation.

Compound	Content, µg/mL
**Anthocyanins**	
Cyanidin-3-O-galactoside (idaein)	382.15 ± 13.19
Cyanidin-3-O-glucoside (chrysanthemin)	31.07 ± 1.10
Cyanidin-3-O-arabinoside	85.87 ± 2.80
Cyanidin-3-O-xyloside	14.35 ± 0.53
**Proanthocyanidin monomers**	
Catechin	40.19 ± 1.33
Epicatechin	322.37 ± 11.75
**Proanthocyanidin dimers**	
EC→EC (1)	171.40 ± 6.23
EC→EC (2)	169.24 ± 6.15
EC→EC (3)	189.86 ± 6.90
EC→EC (4)	157.91 ± 5.74
**Proanthocyanidin trimers**	
EC→EC→EC (1)	225.23 ± 8.16
EC→EC→EC (2)	242.27 ± 8.78
EC→EC→EC (4)	198.92 ± 7.21
EC→EC→EC (4)	249.36 ± 9.04
**Stilbenes**	
trans-Resveratrol-3-O-glucoside	51.92 ± 1.94
**Cyclohexanecarboxylic acid**	
Quinic acid	108.00 ± 4.02
**Hydroxycinnamic acids**	
3-O-Caffeoylquinic acid (chlorogenic acid)	567.06 ± 20.55
Caffeic acid-O-galactoside	98.72 ± 3.58
Caffeic acid-O-glucoside	74.66 ± 2.71
5-O-Caffeoylquinic acid (neochlorogenic acid)	906.08 ± 32.84
p-Coumaric acid-O-glucoside	236.37 ± 8.57
3-O-p-Coumaroylquinic acid	399.47 ± 14.48
Feruloylquinic acid	248.93 ± 9.02
4-O-p-Coumaroylquinic acid	219.83 ± 7.97
Ferulic acid-O-galactoside	131.66 ± 4.77
Ferulic acid-O-glucoside	122.26 ± 4.43
**Flavonol glycosides**	
Quercetin-3-O-rhamnosyl-galactoside	25.57 ± 0.93
Quercetin-3-O-galactoside (hyperoside)	29.17 ± 1.06
Kaempferol-3-O-galactoside	11.15 ± 0.40
Quercetin-3-O-rhamnosyl-glucoside	20.35 ± 0.74
Quercetin-3-O-glucoside (isoquercetin)	22.80 ± 0.83
Kaempferol-3-O-glucoside (astragalin)	9.94 ± 0.36
Quercetin-3-O-arabinoside (guaiaverin)	16.77 ± 0.61
Quercetin-3-O-xyloside	13.97 ± 0.51
Kaempferol-3-O-rhamnosyl-galactoside	12.52 ± 0.45
Kaempferol-3-O-rhamnosyl-glucoside	9.15 ± 0.33
Kaempferol-3-O-arabinoside	11.15 ± 0.40
Kaempferol-3-O-xyloside	12.80 ± 0.46
Total analyzed polyphenols	5840.50

EC–epicatechin; SE FAE–*Sambucus ebulus* L. fruit aqueous extract. Additional data regarding precursor ion and fragment ion mass-to-charge ratios (m/z) of the analyzed polyphenols are given in Table S2. Representative LC-PDA-ESI-MS/MS chromatograms of detected polyphenols are given in Figure S2 (anthocyanins), Figure S3 (proanthocyanidin monomers), Figure S4 (proanthocyanidin proanthocyanidin di- and trimers), Figure S5 (stilbenes), Figure S6 (hydroxycinnamic acids), Figure S7 (hydroxycinnamic acids), Figures S8–S11 (flavonols).

**Table 3:** Oligonucleotide sequences of all used qPCR primer sets.

Gene Name	Forward Primer (5′–3′)	Reverse Primer (5′–3′)
*Actb (β-actin)*	ACGGCCAGGTCATCACTATTG	CAAGAAGGAAGGCTGGAAAAG
*Ptgs2 (COX2)*	TGAGCAACTATTCCAAACCAGC	GCACGTAGTCTTCGATCACTATC
*iNOS*	GGCAGCCTGTGAGACCTTTG	GCATTGGAAGTGAAGCGTTTC
*TNFα*	CCCTCACACTCAGATCATCTTCT	GCTACGACGTGGGCTACAG
*IL-6*	GAGTTGTGCAATGGCAATTCTG	GCAAGTGCATCATCGTTGTTCAT
*IL-1β*	TTCAGGCAGGCAGTATCACTC	CCACGGGAAAGACACAGGTAG
*Ccl2 (MCP-1)*	AGGTGTCCCAAAGAAGCTGTA	ATGTCTGGACCCATTCCTTCT
*Sirt-1*	TGATTGGCACCGATCCTCG	CCACAGCGTCATATCATCCAG
*IL-1ra*	GCTCATTGCTGGGTACTTACAA	CCAGACTTGGCACAAGACAGG
*Icam1*	GACCCCAAGGAGATCACATTC	GAAGATCGAAAGTCCGGA
*Noxo1*	AGAGGAGCCCTTATCCCAACC	TGTCCAGAATTTCTTGAGCCTTG
*Fabp4 (aP2)*	AGTGAAAACTTCGATGATTACATGAA	GCCTGCCACTTTCCTTGTG

## Data Availability

The data presented in this study are available in the article.

## Supplementary Materials

The following are available online at https://www.mdpi.com/article/10.3390/plants10112446/s1, Figure S1: Representative chromatogram of analyzed polar compounds (fraction A) by GC-MS technique, Figure S2: Representative LC-PDA-ESI-MS/MS chromatogram of Sambucus ebulus L. fruit anthocyanins (1-Cyanidin-3-O-Galactoside, 2-Cyanidin-3-O-Glucoside, 3-Cyanidin-3-O-Arabinoside, 4-Cyanidin-3-O-Xyloside), Figure S3: Representative LC-PDA-ESI-MS/MS chromatogram of Sambucus ebulus L. fruit proanthocyanidin monomers, Figure S4: Representative LC-PDA-ESI-MS/MS chromatogram of Sambucus ebulus L. fruit proanthocyanidin di-and trimers, Figure S5: Representative LC-PDA-ESI-MS/MS chromatogram of Sambucus ebulus L. fruit stilbenes, Figure S6: Representative LC-PDA-ESI-MS/MS chromatogram of Sambucus ebulus L. fruit hydroxycinnamic acids (1–3-O-Caffeoylquinic acid, 2–Caffeic acid-O-galactoside, 3–Caffeic acid-O-glucoside, 4–5-O-Caffeoylquinic acid, 5–p-Coumaric acid-O-glucoside, 6–3-O-p-Coumaroylquinic acid, 7–Feruloylquinic acid; 8–4 -O-p-Coumaroylquinic acid; 9–Ferulic acid-O-galactoside; 10–Ferulic acid-O-glucoside), Figure S7: Representative LC-PDA-ESI-MS/MS chromatogram of Sambucus ebulus L. fruit flavonols (1-Kaempferol-3-O-arabinoside, 2-Kaempferol-3-O-xyloside), Figure S8: Representative LC-PDA-ESI-MS/MS chromatogram of Sambucus ebulus L. fruit flavonols (1-Quercetin-3-O-galactoside, 2-Quercetin-3-O-glucoside), Figure S9: Representative LC-PDA-ESI-MS/MS chromatogram of Sambucus ebulus L. fruit flavonols (1-Kaempferol-3-O-rhamnosyl-galactoside, 2-Kaempferol-3-O-rhamnosyl-glucoside), Figure S10: Representative LC-PDA-ESI-MS/MS chromatogram of Sambucus ebulus L. fruit flavonols (1-Quercetin-3-O-rhamnosyl-galactoside, 2-Quercetin-3-O-rhamnosyl-glucoside), Figure S11: Representative LC-PDA-ESI-MS/MS chromatogram of Sambucus ebulus L. fruit flavonols (1-Kaempferol-3-O-galactoside, 2-Kaempferol-3-O-glucoside), Figure S12: Original Western blot gels presenting the changes in protein levels of iNOS, peIF2α, ATF6α and CHOP in J774A.1 mouse macrophages pre-treated with increasing concentrations (2.5%, 5%, 10% *v*/*v*) of SE FAE or with SA for 24 h and subsequently stimulated or not with LPS, Table S1: Relative Kovat’s retention index (RI) of analyzed polar compounds (fraction A) presented in Table 1, using GC-MS technique, Table S2: Precursor ion and fragment ion mass-to-charge ratios (m/z) of the analyzed polyphenols using the LC-PDA-ESI-MS/MS technique.
